# *In vitro* activities of antimicrobial combinations against planktonic and biofilm forms of *Stenotrophomonas maltophilia*

**DOI:** 10.3389/fmicb.2023.1186669

**Published:** 2023-06-20

**Authors:** Bo-An Su, Chi-Chung Chen, Hung-Jui Chen, Hsin-Yu Lai, Chia-Hung Tsai, Chih-Cheng Lai, Hung-Jen Tang, Chien-Ming Chao

**Affiliations:** ^1^Division of Infectious Diseases, Department of Internal Medicine, Chi Mei Medical Center, Tainan, Taiwan; ^2^Department of Pharmacy, Chia Nan University of Pharmacy and Science, Tainan, Taiwan; ^3^Department of Medical Research, Chi Mei Medical Center, Tainan, Taiwan; ^4^Department of Health and Nutrition, Chia Nan University of Pharmacy and Science, Tainan, Taiwan; ^5^Department of Bioscience Technology, Chang Jung Christian University, Tainan, Taiwan; ^6^Division of Hospital Medicine, Department of Internal Medicine, Chi Mei Medical Center, Tainan, Taiwan; ^7^School of Medicine, College of Medicine, National Sun Yat-sen University, Kaohsiung, Taiwan; ^8^Department of Medicine, Chi Mei Medical Center, Tainan, Taiwan; ^9^Department of Intensive Care Medicine, Chi Mei Medical Center, Liouying, Taiwan

**Keywords:** *Stenotrophomonas maltophilia*, levofloxacin, fosfomycin, ceftazidime-avibactam, tigecycline

## Abstract

**Objectives:**

To investigate the *in vitro* activity of antibiotic combinations against *Stenotrophomonas maltophilia* isolates and their associated biofilms.

**Methods:**

Thirty-two *S. maltophilia* clinical isolates with at least twenty-five different pulsotypes were tested. The antibacterial activity of various antibiotic combinations against seven randomly selected planktonic and biofilm-embedded *S. maltophilia* strains with strong biofilm formation was assessed using broth methods. Extraction of bacterial genomic DNA and PCR detection of antibiotic resistance and biofilm-related genes were also performed.

**Results:**

The susceptibility rates of levofloxacin (LVX), fosfomycin (FOS), tigecycline (TGC) and sulfamethoxazole-trimethoprim (SXT) against 32 *S. maltophilia* isolates were 56.3, 71.9, 71.9 and 90.6%, respectively. Twenty-eight isolates were detected with strong biofilm formation. Antibiotic combinations, including aztreonam-clavulanic (ATM-CLA) with LVX, ceftazidime-avibactam (CZA) with LVX and SXT with TGC, exhibited potent inhibitory activity against these isolates with strong biofilm formation. The antibiotic resistance phenotype might not be fully caused by the common antibiotic-resistance or biofilm-formation gene.

**Conclusion:**

*S. maltophilia* remained resistant to most antibiotics, including LVX and β-lactam/β-lactamases; however, TGC, FOS and SXT still exhibited potent activity. Although all tested *S. maltophilia* isolates exhibited moderate-to-strong biofilm formation, combination therapies, especially ATM-CLA with LVX, CZA with LVX and SXT with TGC, exhibited a higher inhibitory activity for these isolates.

## Introduction

*Stenotrophomonas maltophilia* is a glucose-nonfermenting gram-negative obligate aerobic bacillus found in aquatic environments (Brooke, 2012, 2021). In hospitals, *S. maltophilia* has been identified in tap water systems, sink drains, showerheads, and even central venous catheters, endoscopes and dialysate (Nyc and Matejková, 2010). Previous studies also demonstrated that contaminated hospital water could be the source of nosocomial *S. maltophilia* outbreaks (Cervia et al., 2008; Guyot et al., 2013). Clinically, *S. maltophilia* causes various infections, including pneumonia, bloodstream infection, endocarditis, urinary tract infection and soft tissue infection. Among them, pneumonia and bloodstream are the most common nosocomial infections (Singhal et al., 2017; Flores-Treviño et al., 2019; Trifonova and Strateva, 2019; Majumdar et al., 2022; Mojica et al., 2022; Tamma et al., 2022; Umar et al., 2022). Although the virulence of *S. maltophilia* might be low, its associated infections can cause high morbidity and mortality (Falagas et al., 2009; Brooke, 2012; De Mauri et al., 2014; Mori et al., 2014; Trifonova and Strateva, 2019). One of the reasons for the high mortality rate is the limited treatment options due to multiple antibiotic resistance (Brooke, 2012; Majumdar et al., 2022).

*S. maltophilia* produces β-lactamases L1 and L2, which generate β-lactams, cephalosporins, and carbapenems resistant (Avison et al., 2001; Hu et al., 2008). Resistance to aminoglycosides is related to the presence of an aminoglycoside acetyl-transferase (Li et al., 2003). Other resistance mechanisms include efflux pumps, low outer membrane permeability and antibiotic-inactivating enzymes (Singhal et al., 2017). All these mechanisms contributed to the antibiotic resistance of *S. maltophilia*. The current recommended regimens for the treatment of *S. maltophilia* infections are based on previous evidence. Although trimethoprim/sulfamethoxazole and levofloxacin were recommended for treating *S. maltophilia* infection (Singhal et al., 2017; Gibb and Wong, 2021), the resistance to these agents had increased gradually (Wu et al., 2012). The presence of biofilm produced by *S. maltophilia* also causes the treatment to become ineffective (Flores-Treviño et al., 2019; Bostanghadiri et al., 2021). A multicenter prospective study showed that 91.7% of strains could form biofilms, especially bloodborne strains (Pompilio et al., 2020). In this study, more resistance to trimethoprim/sulfamethoxazole and levofloxacin was observed for the biofilm form than the planktonic form (Pompilio et al., 2020). Therefore, there is an urgent need to develop an effective antibiotic regimen to treat *S. maltophilia* infection with resistance and biofilm formation.

The aim of this study was to investigate the *in vitro* activity of antibiotics against *S. maltophilia* isolates and to find a suitable antibiotic combination with synergistic effects to combat antibiotic-resistant *S. maltophilia* and its associated biofilm.

## Materials and methods

**Figure 1:**
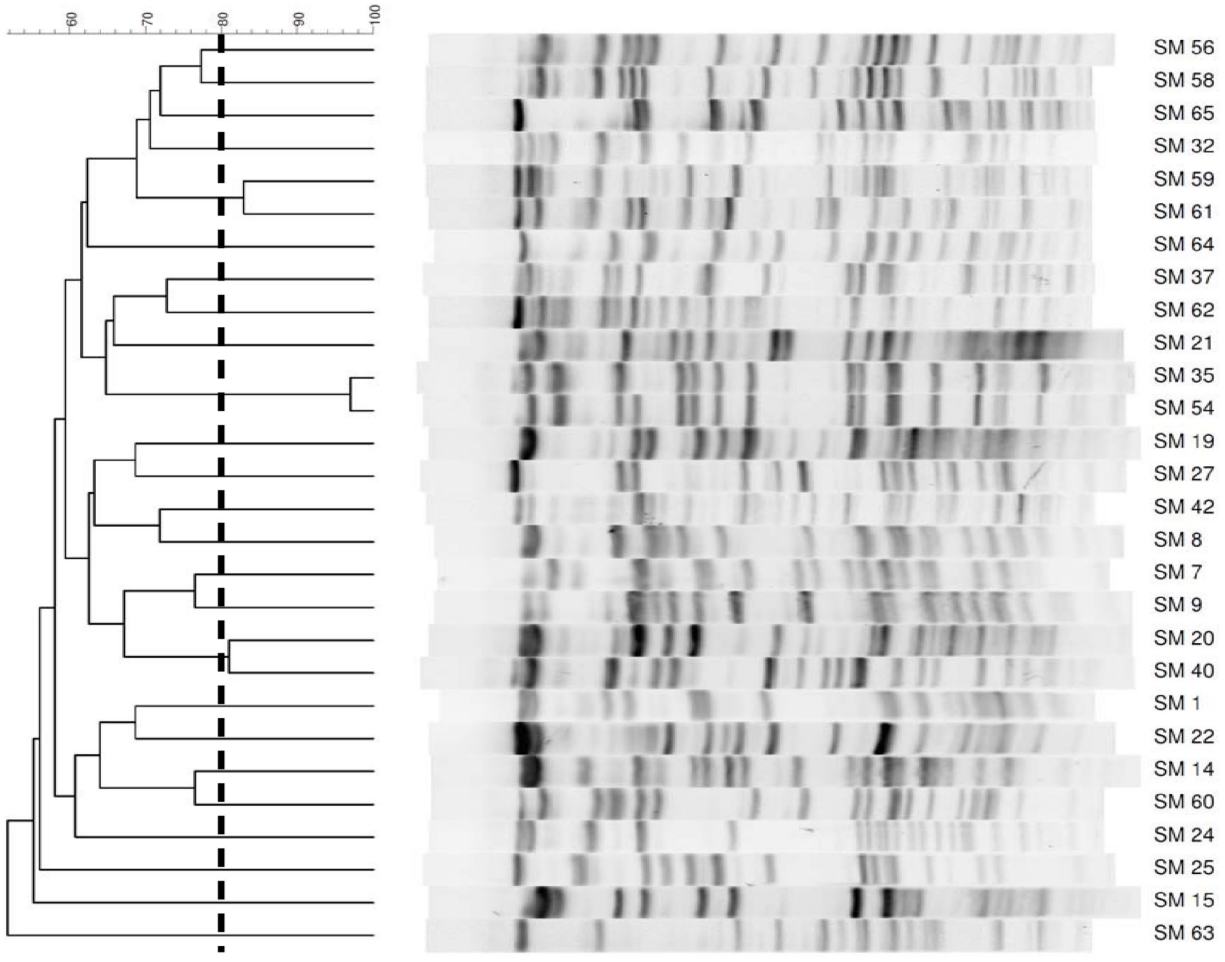
PFGE patterns of 28 *Stenotrophomonas maltophilia* isolates. The number on the scale is the percentage of genetic similarity. PFGE profiles with <80% similarity were considered different. Four strains were untypable.

Thirty-two *S. maltophilia* clinical isolates were collected from the Chi-Mei Medical Center. Species confirmations were performed using matrix-assisted laser desorption ionization time of flight (MALDI-TOF) mass spectrometry (microflex LT, Bruker Daltonics, Bremen, Germany). The isolates were stored at −80°C in Protect Bacterial Preservers (Technical Service Consultants Limited, Heywood, United Kingdom) before use (Chang et al., 2018). The *S. maltophilia* isolates were characterized by PFGE using a CHEF DR II apparatus (Bio-Rad Laboratories, Hercules, CA, United States) with the restriction endonuclease XbaI as described previously (Jumaa et al., 2006). Briefly, bacterial chromosomal DNAs were digested using XbaI (New England Biolabs, Beverly, MA, United States). Electrophoresis was carried out for 22 h at 14°C, with pulse times ranging from 5 to 35 s at 6 V/cm, using a Bio-Rad CHEF MAPPER apparatus (Bio-Rad Laboratories, Richmond, CA, United States). The PFGE patterns were visually examined and interpreted according to the criteria of Tenover et al. (1995). The Dice similarity coefficients were calculated, and PFGE profiles with <80% similarity were considered different pulsotypes. In addition to four strains that were untypable, twenty-five different pulsotypes among 28 isolates were selected for further studies (Figure 1).

### Antibiotics and MIC determination

The antibiotics tested were amoxicillin (AMX), amoxicillin-clavulanate (AMC), aztreonam (ATM), aztreonam-clavulanic (ATM-CLA), chloramphenicol (CHL), ceftazidime-avibactam (CZA), doripenem (DOR), ertapenem, (ETP), fosfomycin (FOS), levofloxacin (LVX), piperacillin (PIP), rifampicin (RIF), sulfamethoxazole-trimethoprim (SXT), tigecycline (TGC), ticarcillin (TIC), ticarcillin-clavulanate (T/C) and piperacillin-tazobactam (TZP). Except avibactam (MedKoo Biosciences, Inc., Morrisville, NC, United States), all drugs were purchased from Sigma (Sigma, St Louis, MO United States). Antibiotic MICs were determined by the agar dilution method and interpretation criteria and were based on the recommendations of the Clinical and Laboratory Standards Institutes (CLSI, 2022) or US Food and Drug Administration (FDA). Specifically, the MIC criteria for CHL, LVX, MIN, SXT, and T/C were based on the guidelines of CLSI for *S. maltophilia*. For AMX, AMC, ATM-CLA, CZA, DOR, ETP, and FOS, the criteria were based on Enterobacteriaceae but for ATM, PIP, TIC, and TZP, the criteria were based on other non-Enterobacterale. For RIF, the criteria were based on *Staphylococcus*. Finally, The criteria for TGC were based on the recommendation of FDA. Briefly, Mueller–Hinton agar (Oxoid, Basingstoke, United Kingdom) was employed to determine the MICs for *S. maltophilia*. Inocula were prepared by suspending overnight cultures in saline to a turbidity equivalent to that of a 0.5 McFarland standard. Inoculated plates were then incubated in ambient air at 37°C for 24 h. Quality control testing was performed using *Escherichia coli* ATCC 25922, *Klebsiella pneumoniae* ATCC 700603 and *Pseudomonas aeruginosa* ATCC 27853 (Shields et al., 2018).

### Minimum concentration for biofilm eradication

The antibacterial activity of each drug in the biofilm was measured using the minimum biofilm eradication concentration (MBEC) assay (Ceri et al., 1999). MBEC indicates the lowest concentrations of the antibiotics added to clear wells in the 96-well ELISA plate. The assay involved biofilm formation on the plastic pegs of the lid of the MBEC device. These biofilms were exposed to antibiotics for 24 h at 37°C, placed in a second 96-well plate containing fresh Mueller–Hinton broth and incubated overnight. The MBEC was the lowest dilution that prevented bacterial regrowth after antibiotic treatment. The MBEC was the lowest dilution that prevented bacterial regrowth after antibiotic treatment. All tests were performed in triplicate. All MBECs of the test drugs were over 1,024 μg/mL (data not shown).

### Biofilm formation

The biofilm formation ability test was performed according to a previous study with modifications (Tang et al., 2012) Briefly, all bacteria were cultured for 1 day at 37°C in 5 mL of tryptic soy broth (Difco Laboratories) with 1% glucose (TSBGlc). The cultures were diluted 1:1000 in TSBGlc, and 200 μL of the solution was added to a 96-well plate. After 24 h of growth at 37°C, the plates were washed three times with PBS to remove unattached bacteria and stained with 100 μL 1% crystal violet (Sigma, St Louis, MO United States). The plate was incubated for 15 min at room temperature; the staining solution was removed, and the plate was washed three times with PBS. After the washing solution was removed, 100 μL of DMSO was added to each well to dissolve the biofilm-bound crystal violet and incubated for 5 min. The OD_570_ was obtained as an index of adherent bacteria and biofilm formation. To compensate for background absorbance, the OD of a sterile medium with fixative and dye was recorded and subtracted from the results. All strains were classified as follows: OD ≤ ODc nonbiofilm formation; ODc < OD ≤ 2 x ODc weak biofilm formation; 2 x ODc < OD ≤ 4 x ODc moderate biofilm formation; 4 x ODc < OD strong biofilm formation (Stepanovic et al., 2000). The experiments were performed in triplicate, and the results are presented as the mean ± SD.

### Killing effects of antimicrobial agents on biofilms after 5 days of treatment

Seven strong biofilm-formation isolates were randomly selected and prepared in 24-well culture plates. These antibiotics (ATM-CLA, CZA, FOS, LVX, SXT, TGC) were used to treat biofilms alone or in combination. Antibiotic concentrations were adjusted to the susceptible breakpoint concentration (SBC) for all tests. The concentrations of antibiotics were adjusted to the SBC for all tests. The SBC was defined according to the CLSI guidelines as 8/4, 2, and 2/38 μg/mL for CZA, LVX, and SXT, respectively, for *S. maltophilia*, and 4/2 and 64 μg/mL for ATM-CLA and FOS, respectively, for Enterobacterales. The SBC for TGC for Enterobacterales was determined to be 2 μg/mL according to recommendation of FDA. The antibiotic-containing medium was gently aspirated after 24 h, and the biofilm was washed with PBS three times. Fresh antibiotic-containing medium was added to the wells continuously for 5 days. To quantify the degree of inhibition of biofilm-embedded bacteria by the tested antibiotics, the biofilms were collected on Day 5. The wells containing biofilms were sonicated using a water-table sonicator for 5 min. The disrupted biofilm was serially diluted, plated and cultured overnight at 37°C, and viable cells were counted. The limitation of detection in this study was 2 log10 CFU/mL. All tests were performed in triplicate (Stepanovic et al., 2000).

### Time-killing effect of biofilms and planktonic bacteria

The biofilms of each isolate were prepared in 24-well culture plates. Antibiotic concentrations were adjusted to the SBC for all tests. The medium in the wells was removed by aspiration, and the biofilm in each plate was treated with sulfamethoxazole-trimethoprim (38/2 μg/mL) or tigecycline (2 μg/mL) at the SBC or sulfamethoxazole-trimethoprim in combination with tigecycline. The other two combinations also received the same experimental treatment. The antibiotic-containing medium was gently aspirated after 24 h, and the biofilm was washed with PBS three times. Fresh antibiotic-containing medium was added to the wells continuously for 5 days. To quantify the degree of inhibition of biofilm-released (planktonic) and biofilm-embedded bacteria by the tested antibiotics, the cell suspension and biofilm were collected on Days 0 (before antibiotic treatment), 1, 3 and 5. The planktonic bacteria were detected using the broth method described above. The wells containing biofilms were sonicated for 5 min. The disrupted biofilm was serially diluted, plated and cultured overnight at 37°C, and viable cells were counted. All tests were performed in triplicate (Shields et al., 2018).

### Antibiotic combination activity assessed by the broth method

The *in vitro* inhibitory effect of combination regimens following the broth killing method was defined in accordance with the CLSI. In brief, the 7 bacterial (as described above) suspensions were diluted to concentrations of 5 × 10^5^ in fresh Mueller–Hinton broth. Three different antibiotic combinations (SXT with TGC, ATM-CLA with LVX, CZA with LVX) were also used in this experiment. Drug concentrations of ATM-CLA, LVX, SXT and TGC were adjusted to 1x MIC, 1/2 x MIC and 1/4x MIC and the SBC for CZA. Bacterial counts were measured at 24 and 48 h; colonies were serially diluted 10-fold in 100 μL aliquots, plated on nutrient agar (Difco Laboratories, Sparks, MD, United States) at 37°C and enumerated (Tang et al., 2021).

### Bacterial genomic DNA extraction and PCR detection of antibiotic resistance genes and biofilm-related genes

The bacterial genomic DNA was extracted using the Bacteria Genomic DNA Kit (Geneaid, Taiwan). The antibiotic resistance determinants and biofilm-related genes were detected by PCR using specific primers. PCR assays were performed using Phusion™ Plus PCR Master Mix (Thermo Scientific), and PCR amplicons were analyzed by 1.5% agarose gel electrophoresis, visualized by health view nucleic acid stain and photographed under UV light (Tang et al., 2021).

## Results

### MIC results

Table 1 shows the MIC results of the tested antibiotics against 32 *S. maltophilia* isolates. No isolates were susceptible to AMX, DOR, ETP, PIP, TIC, and TZP, and less than 20% were susceptible to AMC, ATM, CHL, CZA, RIF, and T/C. A total of 56.3% of isolates remained susceptible to LVX. Additionally, FOS and TGC showed potent activity against 71.88 and 71.88% of isolates, respectively. Finally, SXT was the most active agent, and the overall susceptibility rate was 90.60%.

**Table 1:** MIC results of tested antibiotics for 32 *Stenotrophomonas maltophilia* isolates.

	32 isolates			
	MIC 50	MIC 90	MIC range	Susceptible %
AMX	>128	>128	64 ~ >128	0.00%
AMC	64/32	64/32	4/2 ~ 128/64	6.25%
ATM	>128	>128	4 ~ >128	3.13%
ATM-CLA	8/4	32/16	2/1 ~ 128/64	31.25%
CHL	16	128	4 ~ 128	9.40%
CZA	32/4	>128/4	2/4 ~ >128/4	18.75%
DOR	>16	>16	16 ~ >16	0.00%
ETP	>16	>16	8 ~ >16	0.00%
FOS	64	128	32 ~ >1,024	71.88%
LVX	2	8	0.5 ~ 32	56.30%
PIP	>128	>128	64 ~ >128	0.00%
RIF	8	16	1 ~ 32	6.25%
SXT	0.25/4.75	0.25/4.75	0.25/4.75~ > 16/304	90.60%
TGC	2	8	1 ~ 16	71.88%
TIC	>128	>128	64 ~ >128	0.00%
T/C	128/2	>128/2	2/2 ~ >128/2	12.50%
TZP	>128/4	>128/4	32/4 ~ >128/4	0.00%

AMX, amoxicillin; AMC, amoxicillin-clavulanate; ATM, aztreonam; ATM-CLA, aztreonam-clavulanate; CHL, chloramphenicol; CZA, ceftazidime-avibactam; DOR, doripenem; ETP, ertapenem; FOS, fosfomycin; LVX, levofloxacin; PIP, piperacillin; RIF, rifampin; SXT, trimethoprim-sulfamethoxazole; TGC, tigecycline; TIC, ticarcillin; T/C, ticarcillin-clavulanate; TZP, piperacillin-tazobactam.

### Ability of the isolates to form biofilms

**Figure 2:**
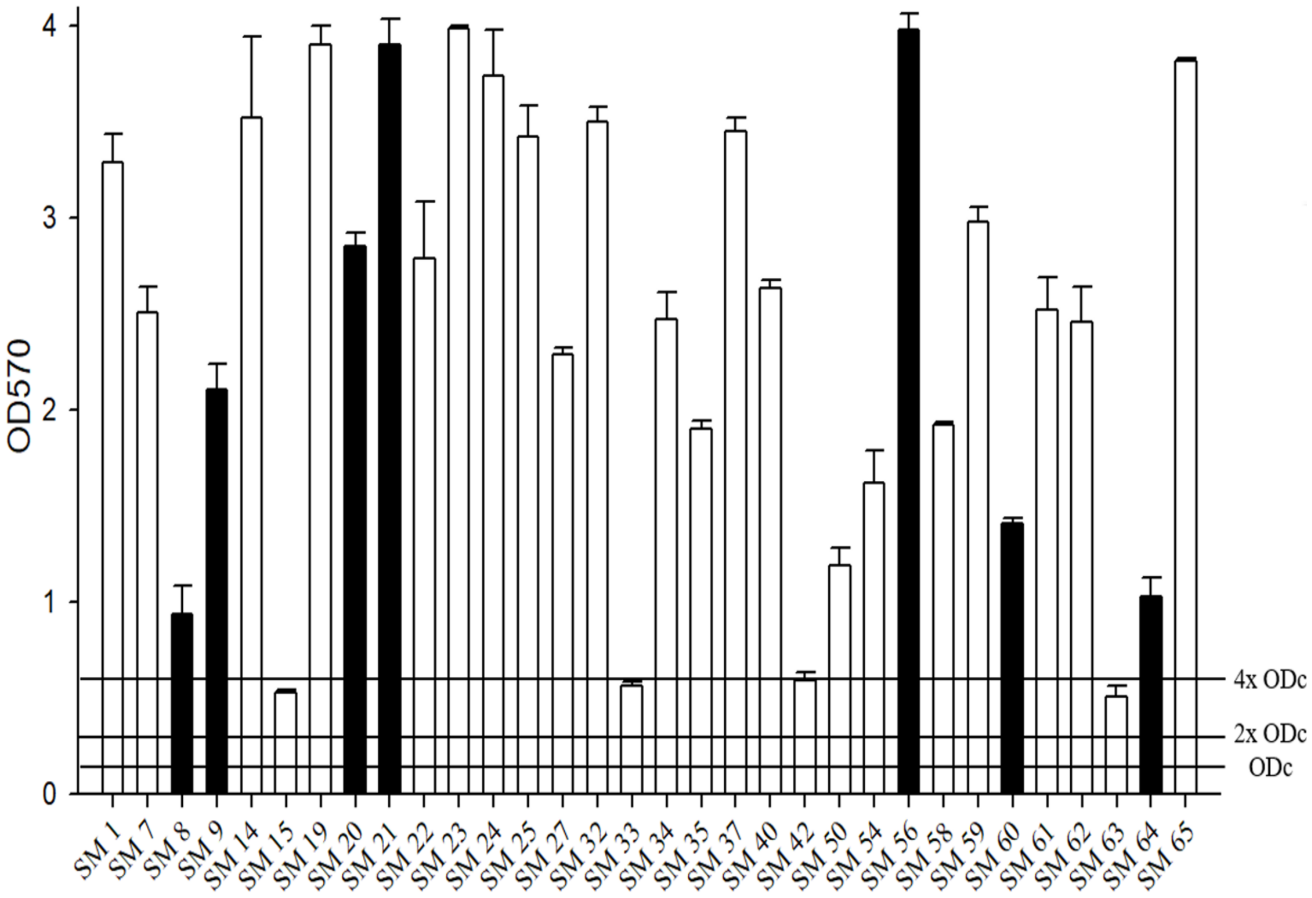
The biofilm formation ability of *S. maltophilia*. The black bars are seven randomly selected biofilm-forming strains for this study. Biofilm formation results of the *S. maltophilia* isolates using the following criteria: OD ≤ ODc nonbiofilm formation; ODc < OD ≤ 2 x ODc weak biofilm formation; 2 x ODc < OD ≤ 4 x ODc moderate biofilm formation; 4 x ODc < OD strong biofilm formation.

All tested isolates showed moderate-to-strong biofilm formation (Figure 2). Among the 32 isolates, four achieved moderate biofilm formation (SM19, SM33, SM42, and SM63), and the others achieved strong biofilm formation.

**Figure 3:**
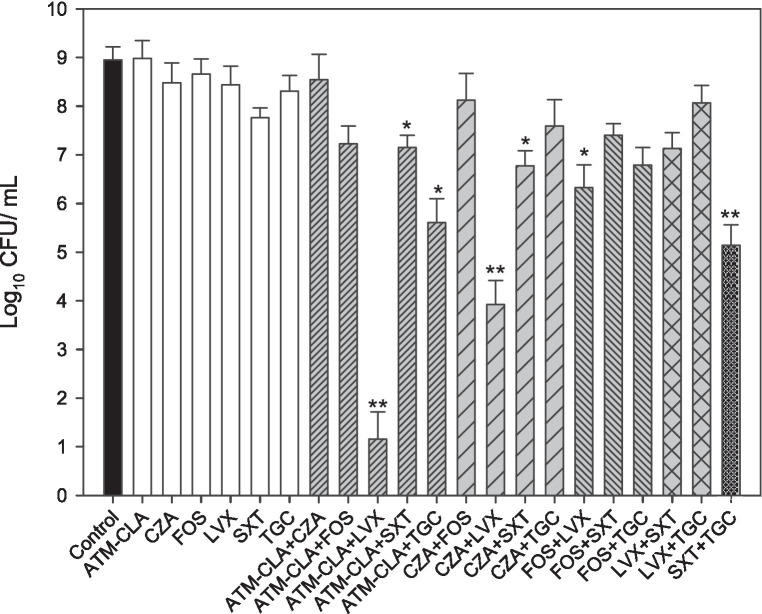
Antimicrobial activities of the six antibiotics used (either alone or in combination) to treat 7 *S. maltophilia* isolates on biofilms. These isolates were exposed to susceptibility breakpoint concentrations of the following drugs for 5 days: 4/2 μg/mL aztreonam-clavulanate (ATM-CLA), 8/4 μg/ml ceftazidime-avibactam (CZA), 64 μg/mL fosfomycin (FOS), 2 μg/ml levofloxacin (LVX), 2/38 μg/mL trimethoprim-sulfamethoxazole (SXT), and 2 μg/ml tigecycline (TGC). Colony counts are shown as the means ± standard deviations. ^*^ *p* < 0.05, ^**^ *p* < 0.01.

Furthermore, seven randomly selected isolates (SM8, SM9, SM20, SM21, SM56, SM60 and SM64) with strong biofilm formation were tested against various antibiotics (ATM-CLA, SXT, LVX, TGC, FOS, and CZA) alone or in combination treatment (Figure 3). Compared to monotherapy, combination therapy (ATM-CLA plus SXT, ATM-CLA plus TGC, CZA plus SXT, FOS plus LVX) exhibited significantly higher activity (all *p* < 0.05). Moreover, the combination with three following regimens -ATM-CLA plus LVX, CZA plus LVX and SXT plus TGC – exhibited more potent activity than that of monotherapy against strong-biofilm formation isolates (all *p* < 0.01).

**Figure 4:**
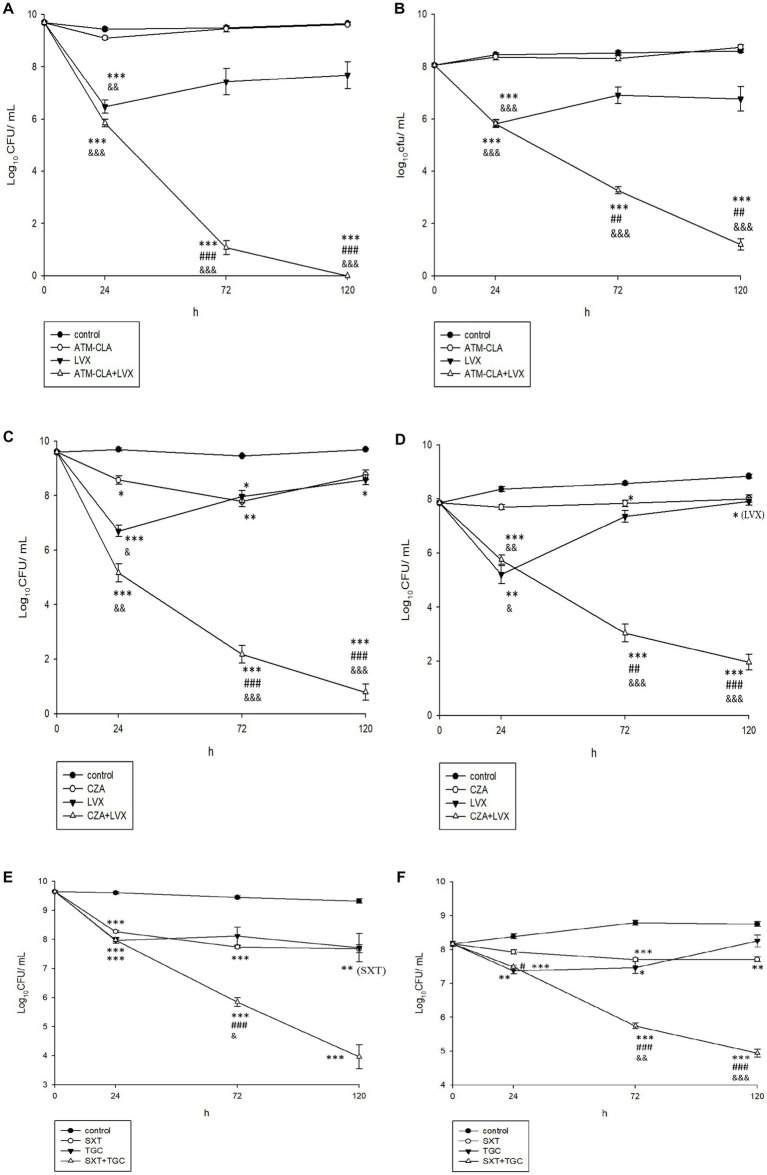
Effect of antibiotic combination on the seven planktonic **(A,C,E)** and **(B,D,F)** biofilm-embedded *S. maltophilia* with strong biofilm formation using time–killing methods with the SBC. *Compared with control. #Compared with the first drug of each figure. &Compared with second drug of each figure. *, # and &: *p* < 0.05. **, ## and &&: *p* < 0.01. ***, ### and &&&: *p* < 0.001.

Using time-killing methods, these three regimens were further tested for their activity on the planktonic and biofilm-embedded *S. maltophilia* with strong biofilm formation (Figure 4). After 72–120 h, ATM-CLA plus LVX exhibited better activity against both the planktonic and biofilm-embedded isolates than that of ATM-CLA or LVX (Figure 4A). Similarly, CZA plus LVX displayed more inhibitory activity than that of CZA or LVX alone after 72 and 120 h (Figures 4A–D). However, the combination with SXT plus TGC only showed better inhibitory against biofilm-embedded isolates than that of SXT or TGC alone, but there was no significant difference between this combination and each component in the inhibitory effect against planktonic isolates at 120 h (Figures 4E,F).

Using broth methods, these three regimens were further tested for their activity on biofilm-embedded *S. maltophilia* with strong biofilm formation (Table 2). After coculture with 1/2x MIC LVX plus 1/2x MIC ATM-CLA at 24 h, they exhibited better activity against biofilm-embedded isolates with a significant decrease in colony compared to that of each drug alone. However, the effect did not persist for 48 h. With 1x MIC LVX plus 1x MIC ATM-CLA, the inhibitory effect persisted for 48 h (*p* < 0.001; Table 2A). With the combination of 1/2x MIC LVX plus 1/2x SBC CZA, the inhibitory effect persisted for 48 h, which was statistically significant compared with each drug alone (*p* < 0.01). With the combination of 1x MIC LVX plus 1x SBC CZA, the inhibitory effect at 48 h persisted more significantly (*p* < 0.001) (Table 2B). After coculture with 1/2x MIC TGC plus 1/2x MIC SXT at 24 h, they exhibited better activity against biofilm-embedded isolates than that of each drug alone. However, the effect did not persist for 48 h. With 1x MIC TGC plus 1x MIC SXT, the inhibitory effect persisted for 48 h (*p* < 0.01; Table 2C).

**Table 2:** The 24- and 48-h killing effect of the antibiotic combination on the seven *S. maltophilia* strains with strong biofilm formation using broth methods with 1x, 1/2x, and 1/4 SBC in ceftazidime-avibactam (CZA) and 1x, 1/2x, and 1/4x MIC in aztreonam-clavulanate (ATM-CLA), levofloxacin (LVX), tigecycline (TGC), and trimethoprim-sulfamethoxazole (SXT).

(A)									
24 h	1x MIC ATM-CLA	1/2x MIC ATM-CLA	1/4x MIC ATM-CLA	Without ATM-CLA	48 h	1x MIC ATM-CLA	1/2x MIC ATM-CLA	1/4x MIC ATM-CLA	Without ATM-CLA
1x MIC LVX	0.77 ± 1.32^***^	3.59 ± 2.13^**^	5.74 ± 1.22	7.06 ± 1.19	1x MIC LVX	1.65 ± 2.26^***^	6.07 ± 2.22^**^	8.24 ± 0.58	8.79 ± 0.56
1/2x MIC LVX	2.54 ± 2.56^**^	6.16 ± 0.98^***^	7.22 ± 0.39^***^	8.44 ± 0.37	1/2x MIC LVX	4.03 ± 3.96^*^	8.58 ± 0.89	8.96 ± 0.10	8.95 ± 0.13
1/4x MIC LVX	5.24 ± 1.79	7.13 ± 0.78	7.82 ± 0.65^***^	8.94 ± 0.11	1/4x MIC LVX	6.13 ± 3.28	8.69 ± 0.81	9.00 ± 0.10	8.98 ± 0.11
Without LVX	6.67 ± 0.84	8.55 ± 0.46	8.89 ± 0.12	9.00 ± 0.14	Without LVX	8.18 ± 0.96	8.98 ± 0.08	9.04 ± 0.12	9.08 ± 0.08

(B)									
24 h	4 ug/mL CZA	2 ug/mL CZA	1 ug/mL CZA	Without CZA	48 h	4 ug/mL CZA	2 ug/mL CZA	1 ug/mL CZA	Without CZA
1x MIC LVX	2.65 ± 2.96^*^	4.78 ± 1.90^**^	5.65 ± 1.35^*^	7.29 ± 1.00	1x MIC LVX	2.51 ± 3.35^***^	5.26 ± 2.69^**^	7.05 ± 1.74^*^	8.62 ± 0.40
1/2x MIC LVX	3.60 ± 3.00*	5.83 ± 1.95^*^	6.87 ± 0.98^*^	8.18 ± 0.61	1/2x MIC LVX	5.66 ± 2.92^*^	7.36 ± 1.12^**^	7.93 ± 1.60	8.96 ± 0.10
1/4x MIC LVX	5.22 ± 3.06	6.61 ± 1.79	7.49 ± 0.80^*^	8.84 ± 0.15	1/4x MIC LVX	6.74 ± 3.13	8.85 ± 0.20	8.44 ± 1.20	9.09 ± 0.10
Without LVX	7.18 ± 1.55	8.06 ± 0.65	8.44 ± 0.57	9.02 ± 0.10	Without LVX	8.47 ± 0.59	8.90 ± 0.18	9.10 ± 0.08	9.12 ± 0.09

(C)									
24 h	1x MIC SXT	1/2x MIC SXT	1/4x MIC SXT	Without SXT	48 h	1x MIC SXT	1/2x MIC SXT	1/4x MIC SXT	Without SXT
1x MIC TGC	4.81 ± 0.79^**^	5.14 ± 0.75^**^	5.21 ± 0.76^**^	6.95 ± 1.04	1x MIC TGC	3.89 ± 1.84^**^	5.18 ± 1.30^***^	5.98 ± 1.89	8.45 ± 1.33
1/2x MIC TGC	6.01 ± 1.00	6.02 ± 1.02^**^	6.35 ± 1.38^**^	8.39 ± 0.46	1/2x MIC TGC	5.85 ± 1.30	6.88 ± 1.36	7.91 ± 1.46	9.12 ± 0.16
1/4x MIC TGC	6.49 ± 0.92	7.19 ± 1.20	7.64 ± 1.06	8.81 ± 0.13	1/4x MIC TGC	6.74 ± 1.50	7.91 ± 1.24	8.88 ± 0.23	8.90 ± 0.21
Without TGC	6.28 ± 0.81	7.64 ± 0.72	8.67 ± 0.43	9.08 ± 0.05	Without TGC	6.44 ± 1.11	8.70 ± 0.55	8.97 ± 0.12	9.10 ± 0.21

Colony counts are shown as the log means ± standard deviations. ^*^ *p* < 0.05; ^**^ *p* < 0.01; ^***^ *p* < 0.001.

### Antibiotic resistance mechanisms

Table 3 summarizes the association between antibiotic resistance genes and the MIC value of the corresponding antibiotics. L1 and L2 β-lactamases were found in 43.8 and 50% of isolates by PCR with L1. L2 full-length primer. Among these isolates, the MICs for carbapenems and β-lactams were especially high. The quinolone-related resistance gene Smqnr was found among 78% (*n* = 25) of the isolates, in which the nonsusceptibility rates to LVX and MOX were only 48.0% (*n* = 12) and 40% (*n* = 10), respectively. Among TMP-SMX-related resistance sul1, sul2, and/or dfrA resistance genes, only three isolates exhibited the sul1 gene, and two of them were resistant to TMP-SMX. Finally, biofilm-related genes spgM, rpfF, and rmlA were detected in 90.6, 53.1 and 81.3% of the studied isolates, respectively (Table 4).

**Table 3:** The association between antibiotic resistance genes and MIC values of corresponding antibiotics.

Isolate	Metallo-β-lactamase			Clavulanic acid-sensitive cephalosporinase		Quinolones			TMP-SMX			TMP		
	L1	DOR	ETP	L2	CAZ	Smqnr	LVX	MOX	Sul1	Sul2	Sul3	dfrA12	dfrA17	TMP-SMX
SM 1	+	>16	>16	−	>128	+	1	0.25	−	−	−	−	−	≤0.25/4.75
SM 7	−	>16	>16	−	64	+	1	0.5	−	−	−	−	−	≤0.25/4.75
SM 8	−	>16	>16	+	128	+	0.5	0.25	−	−	−	−	−	≤0.25/4.75
SM 9	−	>16	>16	+	32	+	2	0.5	−	−	−	−	−	≤0.25/4.75
SM 14	−	>16	>16	+	64	+	4	2	−	−	−	−	−	≤0.25/4.75
SM 15	−	>16	>16	+	>128	+	2	1	−	−	−	−	−	≤0.25/4.75
SM 19	−	>16	>16	+	>128	+	64	8	−	−	−	−	−	>16/304
SM 20	+	>16	>16	+	>128	−	1	0.5	−	−	−	−	−	≤0.25/4.75
SM 21	+	>16	>16	+	>128	+	0.5	0.25	−	−	−	−	−	≤0.25/4.75
SM 22	+	>16	>16	+	>128	−	2	2	−	−	−	−	−	≤0.25/4.75
SM 23	−	>16	>16	+	>128	+	8	4	−	−	−	−	−	≤0.25/4.75
SM 24	+	>16	>16	+	>128	+	2	1	−	−	−	−	−	≤0.25/4.75
SM 25	−	>16	>16	+	>128	−	1	1	−	−	−	−	−	≤0.25/4.75
SM 27	+	>16	>16	+	>128	+	8	4	+	−	−	−	−	≤0.25/4.75
SM 32	+	>16	>16	+	>128	+	2	0.5	−	−	−	−	−	≤0.25/4.75
SM 33	−	>16	>16	−	>128	−	32	16	−	−	−	−	−	≤0.25/4.75
SM 34	−	>16	>16	+	>128	+	8	4	−	−	−	−	−	≤0.25/4.75
SM 35	+	>16	>16	−	>128	+	4	1	−	−	−	−	−	≤0.25/4.75
SM 37	−	>16	>16	+	32	+	1	0.25	−	−	−	−	−	≤0.25/4.75
SM 40	+	>16	>16	+	>128	+	8	8	−	−	−	−	−	≤0.25/4.75
SM 42	−	>16	>16	−	>128	+	8	4	−	−	−	−	−	≤0.25/4.75
SM 50	−	>16	>16	−	8	−	1	0.25	−	−	−	−	−	≤0.25/4.75
SM 54	+	>16	>16	−	>128	+	4	2	−	−	−	−	−	≤0.25/4.75
SM 56	−	>16	>16	−	32	+	1	0.25	−	−	−	−	−	≤0.25/4.75
SM 58	−	>16	>16	−	32	+	1	0.25	−	−	−	−	−	≤0.25/4.75
SM 59	+	>16	>16	−	>128	+	2	0.5	−	−	−	−	−	≤0.25/4.75
SM 60	−	>16	>16	−	16	−	1	0.5	−	−	−	−	−	≤0.25/4.75
SM 61	+	>16	>16	−	>128	+	8	8	+	−	−	−	−	>16/304
SM 62	+	>16	>16	−	>128	+	2	1	−	−	−	−	−	≤0.25/4.75
SM 63	−	>16	>16	−	32	−	32	32	+	−	−	−	−	>16/304
SM 64	+	16	8	−	32	+	4	1	−	−	−	−	−	≤0.25/4.75
SM 65	−	>16	>16	−	128	+	32	32	−	−	−	−	−	≤0.25/4.75

**Table 4:** The association between the biofilm-forming gene and ability (OD ≤ ODc nonbiofilm formation: –; ODc < OD ≤ 2 x ODc weak biofilm formation: +; 2 x ODc < OD ≤ 4 x ODc moderate biofilm formation: ++; 4 x ODc < OD strong biofilm formation: +++).

Isolate	Gene			Ability
	spgM	rpfF	rmlA	
SM 1	+	+	+	+++
SM 7	+	−	+	+++
SM 8	+	−	+	+++
SM 9	−	+	+	+++
SM 14	+	−	+	+++
SM 15	+	+	+	++
SM 19	+	+	+	+++
SM 20	+	+	+	+++
SM 21	+	+	+	+++
SM 22	+	+	+	+++
SM 23	+	−	+	+++
SM 24	+	+	+	+++
SM 25	+	−	−	+++
SM 27	+	−	+	+++
SM 32	+	+	−	+++
SM 33	−	−	−	++
SM 34	+	−	+	+++
SM 35	+	+	+	+++
SM 37	+	−	−	+++
SM 40	+	+	+	+++
SM 42	+	+	+	++
SM 50	−	−	−	+++
SM 54	+	+	+	+++
SM 56	−	−	+	+++
SM 58	+	−	+	+++
SM 59	+	+	+	+++
SM 60	+	−	+	+++
SM 61	+	−	+	+++
SM 62	+	+	+	+++
SM 63	+	−	+	++
SM 64	+	+	−	+++
SM 65	+	+	+	+++

## Discussion

In this study, the antibiotic resistance, biofilm formation and associated mechanisms among *S. maltophilia* were investigated, and several significant findings were obtained. First, *S. maltophilia* remained highly resistant to most antibiotics, including AMX, DOR, ETP, PIP, TIC, TZP, AMC, ATM, CHL, RIF, T/C and even the β-lactam/β-lactamase combination ceftazidime-avibactam (CZA). Although LVX monotherapy was among the recommended antibiotics for treating mild *S. maltophilia* infection (Tamma et al., 2022), the MIC level of LVX was high, and the associated susceptibility rate was only 56.3%. These findings were in line with previous surveillance in Taiwan (Jean et al., 2022), in which the susceptibility of 42 *S. maltophilia* isolates was only 58.1, 46.5 and 41.9% for LVX, CZA, and another novel β-lactam/β-lactamase combination – ceftolozane/tazobactam (C/T), respectively. Another study in Taiwan also showed that the MIC_90_ values of both CZA and C/T were greater than 64 mg/L for 100 *S. maltophilia* isolates (Hsueh et al., 2019). In contrast, the SENTRY Antimicrobial Surveillance Program in the US and Europe reported that 82.5% of 338 *S. maltophilia* isolates remained susceptible to LVX (Shortridge et al., 2022), and another global survey showed that 76.4% of 1,210 *S. maltophilia* isolates were susceptible to LVX (Morrissey et al., 2020). All these findings indicated that the *in vitro* activity of LVX against *S. maltophilia* could differ in different regions/sites and emphasized the importance of continually monitoring and surveilling antibiotic resistance. In Taiwan, the *in vitro* activity of the abovementioned antibiotics, including LVX and β-lactam/β-lactamase combinations, was not potent enough to treat *S. maltophilia* infections.

In addition, we found the potent activity of TGC against *S. maltophilia*. Our findings regarding TGC were consistent with the global surveillance by Morrissey et al., in which the MIC_50_ and MIC_90_ of TGC were 0.5 and 4 mg/L, respectively (Morrissey et al., 2020). In Taiwan, Hsueh et al. obtained similar findings in that the MIC_50_ and MIC_90_ of TGC were as low as ≤0.25 and 2 mg/L, respectively (Hsueh et al., 2019). All these findings indicated the potent *in vitro* activity of TGC; however, further research is needed to assess the clinical efficacy of TCG in the treatment of *S. maltophilia* infection.

In this study, we found that SXT exhibited the most potent *in vitro* activity, with a more than 90% susceptibility rate. This finding was consistent with previous studies (Morrissey et al., 2020; Shortridge et al., 2022), in which the SXT-susceptible rate of *S. maltophilia* isolates ranged from 84.3 to 97.9%. In contrast, the percentage of *S. maltophilia* isolates resistant to SXT significantly increased from 29.7% in 2005–2009 to 47.1% in 2010–2014 (Hu et al., 2018). Overall, SXT remains a good therapeutic option for the treatment of *S. maltophilia* infection in Taiwan and other regions except China.

The *in vitro* activity of the old drug FOS against *S. maltophilia* has rarely been investigated (Lu et al., 2011; Khan et al., 2014). Khan et al. reported that one of two *S. maltophilia* strains causing urinary tract infection was susceptible to FOS (Khan et al., 2014). Lu et al. showed that 59% of 100 *S. maltophilia* isolates were susceptible to FOS, according to the CLSI criteria (Lu et al., 2011). In this study, we found that FOS exhibited potent *in vitro* activity against *S. maltophilia*. All these findings may suggest the potential role of FOS in treating *S. maltophilia* infections.

In line with previous reports (Brooke, 2012, 2021; Santos Carvalhais et al., 2021; Strateva et al., 2023), we observed that all of the tested *S. maltophilia* isolates exhibited moderate-to-strong biofilm formation, which could further increase their resistance to antimicrobial challenge. To overcome this complicated situation, we assessed the activity of various antibiotic combination regimens against seven *S. maltophilia* isolates with strong biofilm formation to find possible solutions. In this study, we tested the inhibitory effect of different combination regimens using time-killing and broth methods and found that compared to monotherapy, combination therapies, especially ATM-CLA plus LVX, CZA plus LVX and SXT plus TGC, exhibited higher inhibitory activity against these isolates. However, the synergistic effect of combination therapy was no consistently observed. Wang et al. reported that compared to azithromycin or fluoroquinolone (FQ) alone, a combination of azithromycin and FQs significantly reduced the biofilm-inhibiting effect against *S. maltophilia* preformed biofilms (Wang et al., 2016). Furthermore, the present finding based on *in vitro* study cannot be directly translated to clinical responses. A retrospective study of 255 patients with *S. maltophilia* pneumonia showed that there was no significant difference in terms of clinical efficacy and resistance development between combination therapy and monotherapy (Shah et al., 2019). Further clinical study is warranted.

Although the common antibiotic-resistant mechanisms were assessed in this study, we found that the prevalence of the antibiotic resistance gene was lower than Bostanghadiri’s et al. (2019) study in Iran. In addition, we observed that the resistant gene cannot be totally translated to antibiotic resistance in the *in vitro* study. Like previous study (Yinsai et al., 2023), we found that the L1/L2 β-lactamase, which were associated the resistance to clavulanic acid and hydrolyses carbapenems, cephalosporins, penicillin and aztreonam (Chang et al., 2015; Bostanghadiri et al., 2019) and sul1 genes were correlated with resistance to β-lactam and TMP-SMX. In contrast, less than half of the isolates with Smqnr, which was encoding the pentapeptide repeat protein that protects both topoisomerase IV and gyrase from the quinolones (Sanchez et al., 2009; Chang et al., 2015; Kanamori et al., 2015; Bostanghadiri et al., 2019) were found to be susceptible to LVX and MOX, which was consistent with the findings of previous studies (Bostanghadiri et al., 2019; Ebrahim-Saraie et al., 2019). Similarly, the biofilm-related genes spgM, rpfF, and rmlA (Bostanghadiri et al., 2021) were not related to biofilm formation ability, which was also compatible with previous reports (Pompilio et al., 2011). Although some target genes might be missing in this study due to mutations in the primer-binding sites associated with new sequences (Mojica et al., 2019), our findings indicated that the antibiotic resistance phenotype might not be fully caused by these antibiotic-resistance or biofilm-formation genes, suggesting that further research is warranted.

In conclusion, *S. maltophilia* remained resistant to most antibiotics, including LVX and β-lactam/β-lactamases; however, the activity of TGC, FOS and SXT remained potent. Although all of the tested *S. maltophilia* isolates exhibited moderate-to-strong biofilm formation, combination therapies, especially ATM-CLA plus LVX, CZA plus LVX and SXT plus TGC, exhibited a higher inhibitory activity for these isolates. Finally, the antibiotic resistance phenotype might not be fully caused by the common antibiotic-resistance or biofilm-formation gene.

## Data availability statement

The original contributions presented in the study are included in the article/Supplementary material, further inquiries can be directed to the corresponding author.

## Author contributions

B-AS, C-CC, and H-JT contributed to conception and design of the study. C-CC, H-JC, H-YL, C-HT, C-CL, and C-MC organized the database. C-CC, H-JC, H-YL, and C-HT performed the statistical analysis. B-AS, C-CC, and CCL wrote the first draft of the manuscript. H-JT and C-MC wrote sections of the manuscript. All authors contributed to the article and approved the submitted version.

## Funding

This study was supported by Chi-Mei Medical Center Research Foundation (CLFHR11111 and CMFHT11202).

## Conflict of interest

The authors declare that the research was conducted in the absence of any commercial or financial relationships that could be construed as a potential conflict of interest.

## Publisher’s note

All claims expressed in this article are solely those of the authors and do not necessarily represent those of their affiliated organizations, or those of the publisher, the editors and the reviewers. Any product that may be evaluated in this article, or claim that may be made by its manufacturer, is not guaranteed or endorsed by the publisher.
